# P300 Aberration in First-Episode Schizophrenia Patients: A Meta-Analysis

**DOI:** 10.1371/journal.pone.0097794

**Published:** 2014-06-16

**Authors:** Yao-qin Qiu, Yun-xiang Tang, Raymond C. K. Chan, Xin-yang Sun, Jia He

**Affiliations:** 1 School of Nursing, Second Military Medical University, Shanghai, P.R. China; 2 Department of Medical Psychology, Faculty of psychology and mental healthy, Second Military Medical University, Shanghai, P.R. China; 3 Neuropsychology and Applied Cognitive Neuroscience Laboratory, Key Laboratory of Mental Health, Institute of Psychology, Chinese Academy of Sciences, Beijing, P.R. China; 4 Department of Statistics, Faculty of Medical Services, Second Military Medical University, Shanghai, P.R. China; University of California, San Francisco, United States of America

## Abstract

**Background:**

Decreased P300 amplitude is one of the most consistent findings in patients with schizophrenia. However, whether prolonged P300 latency occurs in patients with schizophrenia, especially first-episode schizophrenia (FES) patients, remains controversial.

**Methods:**

A meta-analyses of P300 aberration in FES patients and healthy control(HC) group was conducted. The meta-regression analysis was performed using a random effects model. The pooled standardized effect size (PSES) was calculated as the division of the difference between the means of the two groups by the common standard deviation.

**Results:**

A total of 569 FES patients and 747 HCs were included in this meta- analysis. P300 amplitude was significantly reduced (PSES = −0.83, 95% CI: −1.02–0.65, P = 0.00001) and P300 latency was delayed significantly in FES patients (PSES = −0.48, 95% CI: 0.14–0.81, P = 0.005). The meta-regression analysis showed that task difficulty was a source of heterogeneity.

**Conclusions:**

The meta-analysis confirms that disrupted information processing is found in FES patients, which is manifested by smaller P300 amplitude and delayed P300 latency.

## Introduction

Schizophrenia is a serious mental disorder manifesting positive psychotic symptoms such as hallucination and delusion, negative symptoms such as poor motivation and anhedonia, as well as neurocognitive deficits in more than 75% of the patients [1]. Neurocognitive abnormalities may represent a trait marker for schizophrenia [2].

Event-related potentials (ERPs) are cerebral responses associated with various psychological events and are objective parameters reflecting cognitive functions [3]–[4]. The P300 ERP component is a late cognitive-related ERP component associated with attention and memory processes [4]. The P300 event-related brain potential is an index of endogenous cognitive processes that include directed attention and the contextual updating of working memory [5]. The P300 amplitude is viewed as a measure of central nervous system activity that occurs when stimulus memory representations are generated. It is proportional to the amount of attentional resources devoted to a given task [6]. The P300 latency is considered to be a measure of stimulus classification speed. It is unrelated to response selection processes and independent of behavioral response time [7].

P300 deficits have been reported in many mental disorders, such as Alzheimer disease [8], bipolar disorder [9], depression [10], personality disorder [11], and schizophrenia. Previously, a number of studies reported aberrations in both the P300 wave amplitude and the P300 latency in schizophrenia patients. Jeon and Polich reported P300 abnormality of schizophrenia in a meta-analysis research, containing literature published from 1966 to 1999 [12]. After that, Bramon et al. conducted two meta-analyses of P300, respectively in schizophrenia patients [13] and in patients' relatives [14]. Decreased P300 amplitude and prolonged latency were reported in schizophrenia patients and their relatives. Yet subjects included in former meta-analyses were medicated schizophrenia patients, whose P300 might have been affected by antipsychotics. Whether similar abnormalities are also present in medication free first-episode schizophrenia (FES) patients remains unclear. Until now, the effect of medication on P300 amplitude and latency is still a debated issue. Although some research suggested that antipsychotics, especially the second generation agents, could partially improve the performance of P300 amplitude and latency [15], Jeon and Polich did not find any correlation between medication and P300 amplitude effect size in his meta-analysis [12]. Another factor that impacted P300 latency effect size was the disease duration [12]. Delayed P300 latency found in chronic schizophrenia patient may be inconformity with that in FES patients. Until now, there has been no consistent conclusion on P300 latency changes in patients with FES. Although some researchers reported significantly delayed P300 latency in patients [16]–[17], others reported inconsistent results [18]–[20].

This paper aims to conduct a meta-analysis of the studies on P300 in FES patients and to verify the P300 changes in the relatively early stage of schizophrenia.

## Methods

### 1 Study identification

The meta-analysis was designed and reported in line with the PRISMA (Preferred Reporting Items for Systematic Reviews and Meta-Analyses) statement (www.prisma-statement.org). The studies included in this meta-analysis were identified by computerized searching in PubMed, Medline, Cochrane Library, CBM, Chinese sci-tech periodical full-text database, and Full text Chinese academic conference paper database. We searched the databases for papers published between the year 1990 and 2012, using the combined key words “P300” or “P3” or “P3a” or “P3b” and “first episode schizophrenia” or “FES”.

Studies were included if:

- They were published as research papers. Conference abstracts were not included.
- They included human subjects.
- They compared FES patients with a healthy control (HC) group.
- They used the diagnostic criteria of DSM-IV (Diagnostic and Statistical Manual of Mental Disorders 4^th^) or ICD-10 (WHO International Classification of Diseases).
- They reported both mean and standard deviation of amplitude and/or latency of the P300 waveforms. If means and variances were not reported, we requested them from the authors.
- They used a standard auditory or visual oddball task for the P300.
- They reported data at electrodes of Pz and Cz (only those sites were included in the meta-analysis) [13].

Studies were excluded if:

- They were published as abstracts or reviews.
- They had a small sample size (less than seven).
- There were insufficient data on the mean and standard deviation of the P300 amplitude and latency, or the data could not be obtained after two attempted contacts with the authors.
- They used a non-standard auditory P300 paradigm (eg. Speech sounds [21]–[24]).

**Figure 1:**
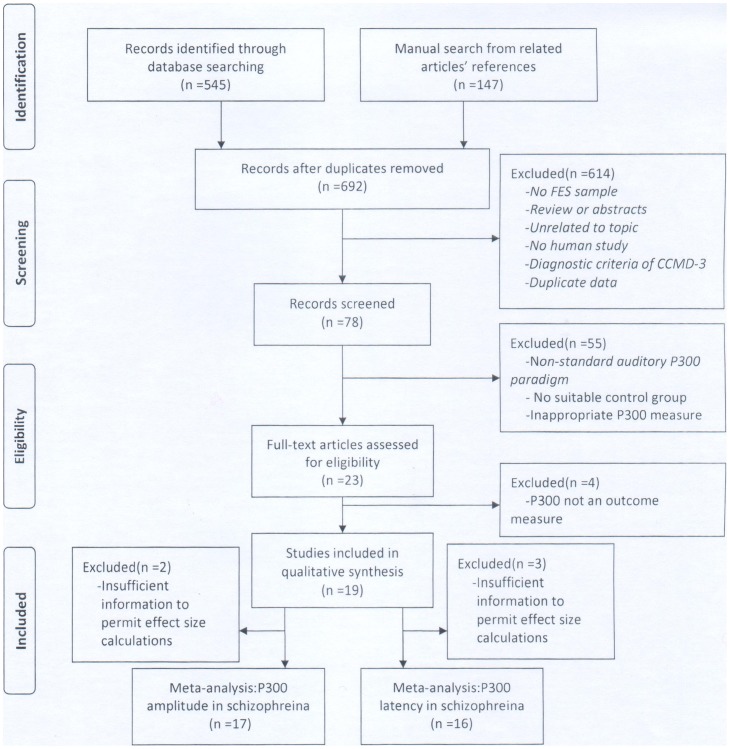
Flow chart of the study identification in the meta-analysis.

Two investigators worked independently and in duplicate, scanned all abstracts and obtained full text reports of the records. After obtaining full text reports of the candidate studies (either in full peer-reviewed publication or press article), the same reviewers independently assessed the eligibility of the full text papers. According to the above criteria, 17 articles were included in this meta-analysis (The working flow chart of the study identification is shown in Fig. 1). Among them, 13 were in English and four in Chinese. Two reviewers conducted data extraction independently using a standardized pre-piloted form. They collected information of the authors, study sites, diagnostic standards, clinical symptom gradings, stimulus parameters, and results. Some of the studies reported P300 measures on both Cz and Pz locations where the P300 values are usually the largest, so that separated effect sizes could be calculated for each of the two electrodes to maximize analysis sensitivity in the same manner. We entered the data into an electronic database such that the duplicate entries of each study could exist. When the two entries did not match, we reached a consensus through discussions.

### 2 Data analysis

In this study, we report our results in standardized effect size (Z scores). Based on the data reported in each study, the Z scores are calculated as the division of the difference between the means of P300 amplitude/latency of the two groups by the pooled standard deviation. The direction of the effect size is negative if the P300 amplitude of FES patients is smaller or if the P300 latency of FES patients is shorter than that of healthy controls. If a study includes one control group and several patient groups, the patient groups are pooled together and their weighted mean is used for analysis as indicated below:
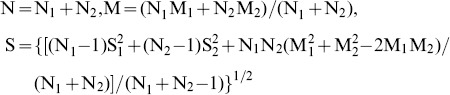
Where,

N = number of pooled number, N_1_, N_2_ = Number of study A and B, M = Weighted mean, M_1_, M_2_ = Mean of study A and B, S = Weighted standard deviation, S_1_, S_2_ = Standard deviation of study A and B.

Most studies reported P300 data at both P_Z_ and C_Z_ electrodes. However, some studies reported data only at one of the two locations. To avoid duplication of analysis and to make use of all available studies, some authors chose the mean data between Pz and Cz for meta- analysis [13]. Considering that the application of the average of amplitude and latency may cause more bias, especially for P300 latency, we entered the larger P300 amplitude and the more delayed P300 latency at P_Z_ and C_Z_. Therefore in our study, the standardized effect sizes were analyzed with random effects meta-analysis.

The random effects regression was used to investigate whether the variability in effect size among the studies could be explained by the variability in study parameters. The following eight predictors were tested: sample size, sex(percentage of male patients), age(mean of patient group), task difficulty(difference between target and non-target), medication(patients were medicated or not), reaction methods(key pressing or counting of targets), target ratio, and study sites (in Chinese or any other country).

We examined the publication bias with the Begg's adjusted rank correlation test and its corresponding funnel plots. A test of heterogeneity (*I* ^2^) among the study results was also carried out.

Heterogeneity test(*I* ^2^), merging effects, subgroup analysis and sensitivity analysis were calculated by Revman 5.0 (http://ims.cochrane.org/revman/download). Publication bias and meta-regression were conducted using STATA 10.0 (Stata Corporation, College Station, TX, USA).

## Results

A total of 78 articles were found by searching the databases with the predefined key words. Among them, three used meta-analysis, 37 were published in Chinese, and the rest in English. After a preliminary screening, 44 articles entered the intensive screening stage. A blind literature quality score was given independently by two readers and the comprehensive evaluation was made by the researchers. In the end, 17 studies on P300 amplitude and 16 studies on P300 latency were suitable for our meta-analysis. For easy understanding, the P3b is from here on referred to as ‘P300’. Among the 17 studies on P300 amplitude, 13 were written in English and 4 in Chinese. These included a total of 569 FES patients (355 males and 214 females) and 747 HCs (424 males and 323 females) [16]–[20], [25]–[36]. The main results of the meta-analysis are summarized in Table 1. As the data of P300 latency could not be acquired from the authors of Liu's study [36], only 16 studies were included in the meta-analysis of P300 latency, with a total of 506 FES (322 males and 184 females) and 707 HCs (405 males and 302 females). In all these meta-analyses on P300 amplitude and P300 latency, 2 researches (Lee et al. 2010 and Wang Qiang et al. 2004) used visual stimuli while the rest used auditory stimuli.

**Table 1:** Studies on the P300 wave included in this meta-analysis.

Author(published year)	Study site	Subjects (FES/HC)	Diagnostic standard	Medication	Clinical symptom grading	Stimuli of paradigm	Reaction methods	High-pass/low-pass filter set(Hz)	Non-target/target frequency(Hz)	Target ratio(%)	Sound (Db)	ISI(s)	Pz/Cz
Ozgürdal(2008)	Germany	31/54	ICD-10	on	21.06±3.08▴	Auditory	Key press	0.16/70	500/1000	23.8	83	1.5	Pz
Brown(2002)	Australia	40/40	DSM-IV	on	75.2±15.1▴	Auditory	Key press	0/50	1000/1500	13.9	60	1.3	both
Hirayasu(1998)	Japan	47/73	DSM-III-R	on	34.26±13.14*	Auditory	Counts	0.16∼30	1000/2000	20.0	75	1.7	Pz
Wang(2010)	China	19/25	ICD-10	off	62.0±10.4▴	Auditory	Key press	0.5/15	1000/1500	20.0	80	1.5∼2.5	both
Korostenskaja(2006)	Lithuania	9/9	ICD-10	off	97.4±14.9▴	Auditory	Counts	0.15∼30	1000/2000	20.0	60	1.5	Cz
Wang(2003)	Japan	20/23	DSM-IV	off	38.9±6.4*	Auditory	Counts	0.16∼30	1000/2000	20.0	75	1.7	both
Salisbury(1998)	USA	14/14	DSM-III-R	on	36.0±4.7*	Auditory	Counts	0.15/40	1000/1500	15.0	97	1.2	both
Demirapl(2002)	Turky	12/12	DSM-III-R	on	60.7±16.5*	Auditory	Counts	0.1/70	1000/2000	20.0	80	2.0	both
Devrim-Ucok(2006)	Turky	30/36	DSM-IV	on	66.4±17.3*	Auditory	Counts	0.5/70	1000/1500	20.0	80	2.0	both
Lee(2010)	South Korea	21/16	DSM-IV	on	18.3±11.4#27.3±14.2▵	Visual	Key press	0.15/40	-	28.1	-	2.7	both
Chen(2007)	China	66/92	DSM-IV	off	111.5±10.9▴	Auditory	Counts	-	500/2000	20.0	80	-	Cz
van der Stelt(2005)	USA	10/14	DSM-IV	on	48.1±4.7▴	Auditory	Key press	0.15/70	1000/1064	8.5	85	1.3∼1.7	both
Umbricht(2006)	Switzerland	26/25	DSM-IV	on	9.7±4.5▵	Auditory	Key press	0.1/30	1000/1500	12.5	75	-	Pz
Wang Qiang(2004)	China	42/120	DSM-IV	off	-	Visual	Key press	-	-	20.0	-	0.5	Pz
Wang Chang-hong(2009)	China	61/32	DSM-IV	on	84.79±13.76▴	Auditory	Key press	-	1000/4000	20.0	Non-target: 80 Target:90	-	Cz
Liu Li(2007)	China	63/40	ICD-10	off	≧60▴	Auditory	Key press	-	1000/2000	20.0	72	-	both
Chen Bin(2010)	China	58/108	DSM-IV	off	-	Auditory	-	-	500/2000	20.0	Non-target:80 Target:85	1.0	Cz

Note:

*represents BPRS,

▴ represents PANSS,

# represents SAPS,

▵ represents SANS.

### 1 P300 amplitude and latency in FES patients

There was evidence of significant between-study heterogeneity for both P300 amplitude (*τ* ^2^ = 0.08, *χ* ^2^ = 35.56, df = 16, p = 0.003) and P300 latency (*τ* ^2^ = 0.37, *χ* ^2^ = 103.96, df = 15, p<0.001), which supports the use of random effects meta-analysis method.

**Figure 2:**
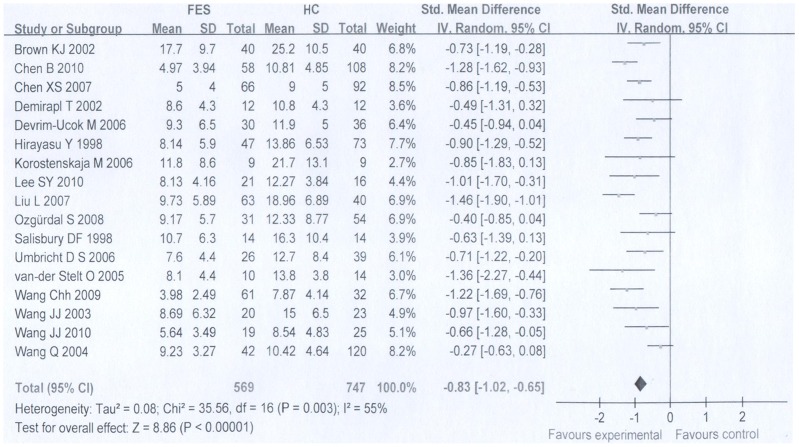
Meta-analysis on the forest map of P300 amplitude of FES group and HC group.

**Figure 3:**
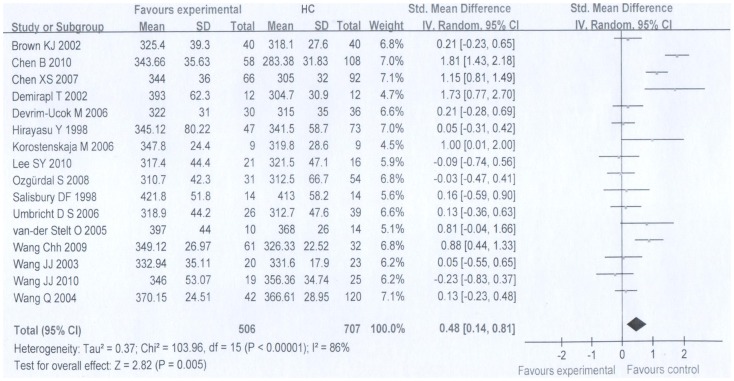
Meta-analysis on the forest map of P300 latency of FES group and HC group.

Random effects meta-analysis demonstrated that when compared with healthy controls, FES patients had significantly smaller P300 amplitude with a pooled effect size of −0.83 (95% CI −1.02 to −0.65, P = 0.00001). The P300 latency was delayed significantly when compared with controls with a pooled effect size of 0.48 (95% CI 0.14 to 0.81, P = 0.005) (Fig. 2, 3).

We found no evidence of publication bias either for P300 amplitude (Begg's test: z = 0.04, P = 0.967) or for P300 latency (Begg's test: z = 0.32, P = 0.753).

### 2 Meta regression

Sex, age, task difficulty, medication, reaction methods, target ratio, and study sites might all influence the P300 waves. Meta-regression analysis showed that task difficulty had a significant influence on the effect sizes of P300 amplitude and P300 latency, which could explain the heterogeneity found in our study. No significant influence of sex, age, medication, reaction methods, target ratio and study sites on heterogeneity.

For P300 amplitude, after “task difficulty” was introduced into the meta-regression model, the heterogeneity (*τ* ^2^) decreased from 0.08 to 0 (see Table 2, 3). Fifteen studies(2 visual oddball studies, Lee(2010) and Wang(2004), were not included in meta-regression)were then divided into two subgroups: a subgroup (7 studies) with high task difficulty (the difference between target and non-target < = 500 Hz) and a subgroup (8 studies) with low task difficulty (the difference between target and non-target >500 Hz). No heterogeneity was found in the two subgroups (*I* ^2^ = 0), and a fixed-effect model was introduced in the meta-analysis. The merging effect of the two subgroups was consistent with each other.

**Table 2:** Meta regression analysis for P300 amplitude.

Variables	Coef.	Std. Err.	t	p>|t|	[95% Conf. Interval]	
sample size	0.128	0.198	0.65	0.542	−0.357	0.614
sex	−0.023	0.227	−0.10	0.921	−0.578	0.531
age	0.090	0.229	0.39	0.708	−0.470	0.650
medication	−0.110	0.185	−0.60	0.572	−0.563	0.342
reaction methods	0.410	0.203	2.02	0.090	−0.086	0.905
task difficulty	−0.671	0.239	−2.80	0.031	−1.256	−0.085
target stimulation ratio	0.243	0.230	1.06	0.332	−0.320	0.805
study sites	0.460	0.239	1.92	0.077	−0.057	0.976

**Table 3:** Meta regression analysis for P300 latency.

Variables	Coef.	Std. Err.	t	p>|t|	[95% Conf. Interval]	
sample size	0.298	0.323	0.92	0.371	−0.394	0.990
sex	0.011	0.338	0.03	0.975	−0.713	0.735
age	−0.162	0.331	−0.49	0.631	−0.872	0.547
medication	0.375	0.327	1.15	0.270	−0.325	1.076
reaction methods	0.288	0.275	1.05	0.313	−0.305	0.881
task difficulty	0.805	0.285	2.82	0.014	0.189	1.420
target stimulation ratio	0.261	0.383	0.68	0.507	−0.561	1.082
study sites	−0.082	0.333	−0.25	0.809	−0.795	0.631

For P300 latency, fourteen studies (2 visual oddball studies, Lee(2010) and Wang(2004), were not included in meta-regression.) were also divided into high and low task difficulty subgroups. Only the high task difficulty subgroup (7 studies) showed a significantly delayed P300 latency in FES patients compared with healthy controls (z = 3.23, p = 0.001), but with a large heterogeneity among the studies (*I* ^2^ = 89%). Compared with HCs, no prolonged P300 latency was found in FES patients in the low task difficulty subgroup(7 studies)(z = 1.10, p = 0.27).

## Discussion

Clinically, schizophrenia patients often present attention, verbal memory, and working memory dysfunctions [37]. Previous studies suggested that aberrations of P300 amplitude in schizophrenia patients were indicators of these dysfunctions [38], which might be further related to structural and functional changes in the schizophrenia patient's temporal lobe and parietal lobe [39]. Although most of the previous studies reported a reduced P300 amplitude at Pz/Cz electrode in FES patients [16]–[20], [25]–[29], [21]–[36], some studies reported negative results [30], [40]. A variety of factors, such as disease severity and sample size, might explain this apparently inconsistent result [12]. Our meta-analysis results revealed that Pz and Cz electrode merging effects of significantly decreased P300 amplitude in FES patients were lower than that of normal normal healthy control groups. Given that the P300 is often viewed as an index of cognitive function [41], our results may suggest that cognitive impairment could manifest itself even in the early stage of illness. Some research suggested that P300 stemmed from a wide cerebral cortex network, such as parietal lobe [42], cingulated gyrus, and frontal lobe [43]. The parietal lobe was considered to related with selective attention [42], while cingulated gyrus and frontal lobe were related to working memory [43]. Our findings suggest that the dysfunction of corresponding brain area may take place in FES patients.

P300 amplitude deficits were considered as an index of disturbances in attentional allocation mechanisms [6]. Recently, a series of neuropsychological studies on attention dysfunction in schizophrenia patients have supported this conclusion. Brébion [44] suggested that selective attention disorder in schizophrenia patients might reduce reality-differentiating capacity, leading to hallucinations and delusions. Besides, another study revealed a positive correlation between negative symptoms severity and attention disorder [45]. In addition, some studies also showed that the attention disorder found in schizophrenia patients might exhibit disease specificity to a certain degree [46]–[47]. Evidence also showed that P300 abnormality occurred not only in schizophrenia patients, but also in their non psychotic relatives [48] and ultra high risk (UHR) subjects [49].

In recent years, a number of longitudinal studies on the change of P300 amplitude in schizophrenia patients during different time periods have emerged [15], [50]–[51]. Some researchers think that P300 aberrations might serve as a trait marker for schizophrenia [15], while the others think P300 is a state marker for the patients. However, loss to follow-up [52], high cost of follow-up [13], medication [53], and other factors have made these studies less convincing. Our meta-regression tests revealed no significant effect of medication on P300 measures. This suggests that symptomatic treatment does not alter pathophysiology, the determinant of P300 values. Furthermore, this results suggested that P300 abnormalities may more likely be a ‘trait’ rather than a ‘state’ marker of schizophrenia. Prospective longitudinal studies of first episode patients need to be carried out in order to fully establish which neurophysiological abnormalities are early ‘traits’ and whether they change with disease progression.

Some studies have examined the effect of the physical characteristics on P300 amplitude and latency [54]–[55]. P300 effects of task difficulty was reported by Jeon and Polich in their meta-analysis [12]. In this meta-analysis, we also found that task difficulty had a significant influence on the effect sizes of P300 amplitude and P300 latency. The results of subgroup analysis also confirmed that task difficulty was the main source of heterogeneity. Prolonged P300 latency was only found in the subgroup with small difference between target and non-target stimulus, but not in the subgroup with large stimulus difference. We speculate that, more attention resources would be demanded for small difference P300 test (difficult) than for large difference one (easy). Insufficient resources can be consumed in the difficult test in FES, which may cause smaller P300 amplitude and prolonged P300 latency. When the difference between target and non-target is large, insufficient brain resource was engaged in FES, the same as in the difficult test; while the speed of information processing in schizophrenia patients was not affected. Therefore no significant difference of P300 latency was found between patients and healthy controls.

The observation of a prolonged latency in schizophrenia patients is controversial [56]–[57]. Our meta-analysis results demonstrated prolonged P300 latency in schizophrenia patients relative to healthy controls, which indicated a decline in patients' brain processing speed of external information. This information processing disorder reflects itself not only in P300 changes, but also in other indicators such as mismatch negativity(MMN) [58] and auditory function [59]. Notably, our meta-regression analysis, subgroup analysis, and sensitivity analysis all indicated substantial heterogeneity in merging the effect of P300 latency change in FES patients, with only one exception. One subgroup(low task difficulty) showed no heterogeneity and no statistical significance of P300 latency between FES patients and healthy controls. Consequently, if we want to get more comprehensive explanation of P300 latency variation in schizophrenia, better designed experiments are needed in future research.

One limitation of this meta-analysis is the possibility of publication bias. There was no evidence of publication bias for P300 amplitudes (P = 0.967) and latency(P = 0.753). However, we included only published studies, while certain non-published studies may have been conducted on this topic. We limited our search to English and Chinese language databases, while qualified studies in other languages could exist. When we were conducting meta-regression, two visual oddball studies (Lee(2010) and Wang(2004)), were excluded. In addition, to avoid duplication of analysis, we entered the larger P300 amplitude and the more delayed P300 latency at Pz and Cz. This method may cause some bias in meta-analysis. Besides, some other factors such as EEG recording methodology and clinical severity may also be the source of heterogeneity; however, no sufficient information can be extracted from previous researches. Therefore, at present, it is not possible to explore the effects of these factors.

## Conclusions

It could be concluded from this meta-analysis that FES patients demonstrate obvious aberrations of P300 amplitude and latency. As no significant effect of medication on P300 was found in our meta-regression tests, we infer that these abnormalities may be ‘trait’ markers of schizophrenia. Heterogeneity in subgroups would be greatly reduced when the subgroups are differentiated by task difficulty, and the combined effect of P300 amplitude could be more stable.

## Supporting Information

Checklist S1 PRISMA Checklist.
(DOC)
